# Docosahexaenoic acid

**DOI:** 10.1016/j.advnut.2023.100161

**Published:** 2023-12-02

**Authors:** Caroline Richard, Jennifer M Monk

**Affiliations:** 1 Department of Agricultural, Food and Nutritional Science, University of Alberta, Edmonton, Alberta, Canada; 2 Department of Human Health & Nutritional Sciences, University of Guelph, Guelph, Ontario, Canada

**Keywords:** DHA, Fatty acids, Fish oil, lactation

## Nutrient:

Docosahexaenoic acid (DHA) is a long chain polyunsaturated fatty acid (LCPUFA) of the omega-3 (n-3) family with a 22-carbon chain and six *cis* double bonds (22:6n-3) [1]. DHA is not a dietary essential fatty acid (FA) *per se* since it can be metabolized to some extent from its dietary essential precursor α-linolenic acid (ALA, 18:3n-3) via a series of desaturation and elongation reactions. Although ALA can be metabolically converted into eicosapentaenoic acid (EPA, 20:5n-3) and further converted to DHA, the conversion rate is fairly low in humans [1, 2]. Consequently, plasma and tissue levels of DHA are determined mainly by dietary DHA intake, and consumption of large amounts of ALA has been shown to have little effect on plasma DHA concentrations, except in those with very low dietary DHA intakes [2]. As a result, many organizations worldwide have issued a recommendation for dietary DHA intake (often combined with intakes of EPA) [3].

Most evidence for the health benefits of n-3 LCPUFAs is based on studies of fish consumption and/or fish oil supplementation that contain both EPA and DHA in different proportions. Therefore, we still know relatively little about the unique effects of DHA (i.e., without confounding effects of dietary EPA) on health outcomes. Yet, in recent years, n-3 LCPUFAs formulations containing either EPA or DHA alone have been assessed mainly as they pertain to coronary artery disease (CAD) management in adults [4] and visual/cognitive [2] or immune function [5] in early developmental periods in infants.

Most of the evidence for the benefit of DHA relates to its unique role in cognitive and visual development and function. DHA is found in high concentrations in neuronal cell membrane phospholipids, where it can exert many physiological roles, including regulating membrane fluidity, neurotransmitter release, gene expression, myelination, and cell differentiation and growth [2]. Considering the low rate of *de novo* DHA synthesis from ALA, many organizations agree that DHA is required in the maternal/infant diet in order to reach and maintain adequate DHA levels in the brain and retina to support their functions in the infant [2, 3]. DHA rapidly accumulates in the brain and retina during gestation and early infancy and is essential for infant growth and maturation. Breast milk naturally contains DHA: analysis of breast milk samples from over 2400 females worldwide reported a mean concentration of DHA in breast milk of 0.32 g/100 g of total FA ranging from 0.06 to 1.4 [6]. Breast milk DHA concentrations will vary based on maternal dietary DHA intake levels. Hence, infant formulas are typically enriched in DHA (between 0.2–0.35% of total FA) for optimal brain and visual development in both preterm and full-term infants [6]. Although recent systematic reviews and meta-analyses have failed to report a consistent beneficial effect of higher DHA intakes on cognitive and visual function early in life, most reported a beneficial effect on gestational length and infant birth weight [2]. In adults, whereas n-3 LCPUFAs supplementation has led to some improvement in individuals with mild cognitive impairment, data are inconsistent as to whether they can be beneficial in older adults with health conditions impacting the brain and eye, such as dementia and Alzheimer’s disease or macular degeneration [2]. To the contrary, n-3 LCPUFA supplementation during pregnancy and/or lactation, as well as infant formulas enriched in DHA, have often led to some immune benefits early in life (i.e., decreased risk of allergic diseases and asthma) compared with infants fed formulas devoid of DHA [5].

A large body of evidence from epidemiological and intervention studies points toward an overall cardioprotective effects of EPA+DHA [2, 4]. Meta-analyses of observational and intervention studies have reported that the intake of EPA+DHA dose-dependently reduced risk of some CAD events and mortality from CAD. Several potential mechanisms could be responsible for this lowered risk of mortality, including reduced susceptibility to cardiac arrhythmias, stabilization of atherosclerotic plaques, lowered plasma triglyceride (TG) concentrations, slight reductions in blood pressure, and decreased markers of systemic inflammation and oxidative stress [2, 4]. Yet, recent evidence from intervention studies comparing EPA and DHA suggest that although both LCPUFA can reduce plasma TG levels and exert anti-inflammatory effects, DHA tends to reduce TG and certain markers of inflammation (e.g., IL-18) slightly more effectively compared with EPA, while also modestly increasing low-density lipoprotein-cholesterol (LDL-C; and its particle size) and high-density lipoprotein-C (HDL-C) concentrations compared with EPA [4]. Inflammation is a key etiological factor in the pathogenesis of chronic diseases such as CAD, and incorporation of DHA into cell membranes leads to the generation of anti-inflammatory lipid mediators implicated in the resolution phase of inflammation, such as resolvins, protectins, and maresins [7]. Whereas several observational and intervention studies have reported an anti-inflammatory effect of EPA+DHA, the effects appear to also depend on the health status of populations studied. n-3 LCPUFAs seem to effectively lower inflammation in rheumatoid arthritis, whereas results have been inconsistent for inflammatory bowel disease [2]. Regarding cancer, n-3 LCPUFAs have been shown to reduce risk of breast cancer and potentially colorectal cancer while increasing the sensitivity of tumor cells to conventional therapies without affecting normal cells [2]. Yet, more intervention trials are needed to clarify the role of n-3 LCPUFAs in cancer prevention/treatment.

## Deficiencies:

LCPUFA deficiency is rare; however, deficiency in both essential FAs (n-3 ALA or n-6 Linoleic acids) can lead to scaly skin or dermatitis [1]. Since DHA can be synthesized from ALA, there is perhaps no frank deficiency in DHA; despite the conversion rate of ALA to DHA in humans being fairly low, it has been shown to increase in adults not consuming DHA [1, 2]. Although DHA concentrations in plasma and tissues are modulated by dietary n-3 intake, no cut-off concentrations of DHA for which visual or cognitive function is impaired have been identified to date.

## Diet recommendations:

Due to a lack of evidence in healthy populations, there is currently no specific dietary reference intake (DRI) for DHA. Adequate intake values have been proposed for different groups of the population only for ALA, the dietary essential precursor FA for *de novo* synthesis of EPA and DHA[1]. However, numerous organizations worldwide have issued recommendations for DHA (often combined with EPA) intake and/or for fish intake. The 2020–2025 Dietary Guidelines for Americans and Canada’s Food Guide recommend that the general population, along with pregnant and breastfeeding females, should consume at least 8 to 12 ounces (i.e., 2 servings) of seafood per week providing ≥ 250 mg/d of EPA+DHA. Other health organizations have also provided specific recommendations for combined EPA+DHA intakes across different population groups, ranging, on average, from 250 to 500 mg/d for adults (3). There is also a general agreement that infant formulas should be fortified with DHA (at least 0.32% of total FAs) to provide the average content found in breast milk worldwide [6].

## Food sources:

Seafood and fish are the major dietary sources of DHA, particularly cold-water fatty fish, including salmon, herring, tuna, mackerel, anchovies, and sardines [2]. These fatty fish are by far the richest dietary source of DHA, with each 75 g fish serving providing between 750 and 1500 mg of EPA+DHA. Breast milk also naturally contains DHA, and its concentration increases with higher maternal intakes of EPA+DHA, while most infant formulas in the US are fortified in DHA to support brain development. Nowadays, there is also a variety of food products fortified in fish oil-derived EPA and DHA, including eggs, yogurt, milk, juice, and soy beverages [2]. Finally, many supplements, including fish oil, krill oil, cod liver oil, and algal oil (for vegetarians), exist on the market containing varying doses of EPA and DHA, depending on the supplement.

## Clinical uses:

The American Heart Association (AHA) recommends that individuals with existing CAD take 1 g/d of EPA+DHA for secondary prevention of CAD, wherein both fatty fish consumption and fish oil supplements are recommended to achieve this recommended intake [2, 4]. The AHA also recommends consumption of >3 g/d of EPA+DHA to lower plasma TG levels in individuals with high (200–499 mg/dL) to very high TG (≥500 mg/dL) under a physician’s care [4]. These amounts of n-3 LCPUFA can be achieved with fish oil supplements or prescription formulations approved by the US Food and Drug Administration (FDA) that contain either EPA+DHA (omega-3 acid ethyl esters (O3AEE); Lovaza) or EPA alone (icosapent ethyl (IPE); Vascepa) [4].

## Toxicity and adverse outcomes

Similar to the lack of DRI recommendations, no tolerable upper intake level has been established for ALA or DHA [1]. Although regular intake of DHA (< 2–4 g/d EPA+DHA) is generally considered safe for most healthy individuals, specific groups of the population may be at higher risk of adverse effects. These can include a fishy taste, nausea/eructation, intestinal discomfort (gas, diarrhea/constipation), pruritus/rash, bruising, and increased risk for bleeding [2, 4]. Therefore, individuals taking anticoagulant/antiplatelet medications should be careful when taking high doses of EPA+DHA. There is also a concern about mercury content in fish that can vary depending on the geographic region, and therefore, pregnant and lactating females, as well as young children, are recommended to limit certain types of fish with higher mercury content [2]. Finally, although EPA and DHA do not cause allergic reactions, the FDA recommends caution for individuals with seafood allergies depending on the purity of some fish oil supplements.

## Recent research

Further research is needed to fully elucidate the independent roles of DHA and EPA on health outcomes and establish a specific DRI for EPA+DHA intake for which data from several trials inprogress are anticipated. In particular, results from the DHA WIN trial investigating the role of DHA in conjunction with chemotherapy on breast cancer outcomes are forthcoming. With respect to LCPUFA supplementation during pregnancy, recent evidence from randomized control trials has shown improved cognitive development in both infants and children; however, the evidence supporting the beneficial effects of DHA on other neurodevelopmental outcomes is less consistent or insufficient [8]. Additional longitudinal studies assessing the impact of LCPUFA intakes during pregnancy and lactation as well as early in life during infancy on a spectrum of neurodevelopmental outcomes (e.g., social-emotional, visual, motor, language development, etc.) are required. A recent study assessing FA intakes in preschool-aged children (mean age 3.6 y) demonstrated that 74.3% had EPA+DHA intakes below recommended intakes [9], thereby highlighting the need to assess both maternal and child LCPUFA intakes throughout critical neurodevelopmental stages and extending into adolescence.

## Further information

For more information, please refer to these recently published reviews [2, [4], [5], [6], [7], [8]] and the Dietary Reference Intakes guide [1].

## Authors contributions

The authors’ responsibilities were as follows—CR wrote the manuscript, which was reviewed by JM. CR has primary responsibility for the final content. Both authors have read and approved the final manuscript.

## Conflict of interest

C. R. reports no conflict of interest. J. M. reports no conflict of interest.

## Funding

C.R. is supported by the Canada Research Chair program (CRC-2018-00081).
